# Literary Notes

**Published:** 1899-08

**Authors:** 


### LITERARY NOTES.

THE *Bulletin* of the Cleveland General Hospital is a quarterly magazine of medical science, edited by Dr. Charles J. Aldrich for the staff of the Cleveland General Hospital. It is a handsomely printed, well edited, magazine and is in every way a credit to all concerned in issuing it. It is a pity, however, that it is not sent out flat. In these days the usual receptacle of the rolled journal is the waste basket; but it would be a mistake under such a rule to consign this handsome magazine to such base uses.

---

WILKINSON'S *Medical Digest* is the name of a new publication which takes the place of the Omaha *Clinic*, that was formerly edited by Dr.

Wilkinson. The *Digest* contains good material, but it is ungainly in size. We think it a mistake for medical publications to aim to be *outré*, either in appearing under or over the ordinary size.

---

THE Louisville *Journal of Medicine and Surgery* and the *Louisville Medical Monthly* have been consolidated and the new magazine will be issued under the name of the *Louisville Monthly Journal of Surgery and Medicine.* It will continue to be edited by Professor Joseph M. Mathews, M. D., LL.D., and Dr. Horace H. Grant. Dr. Grant will be the business editor and Dr. Henry E. Tuley, former editor of the *Monthly,* will be managing editor of the newly consolidated magazine. The editorial staff will be further reinforced by the addition of Dr. A. M. Cartledge, an accomplished physician and a well-known writer. Certainly this is a strong combination of medical talent, and it ought to, as it no doubt will, make the new enterprise one of the best medical journals in the country.

---

MR. W. B. SAUNDERS, medical publisher, of Philadelphia, announces the following list of books to be published on or before September 1, 1899: The International Text-book of Surgery. In two volumes. By American and British authors. Edited by J. Collins Warren, M. D., LL.D. A Text-book of Embryology, by John C. Heisler, M. D. Diseases of the Nose and Throat, by D. Braden Kyle, M. D. The Treatment of Pelvic Inflammations through the Vagina, by W. R. Pryor, M. D. The Hygiene of Transmissible Diseases: their causation, modes of dissemination and methods of prevention, by A. C. Abbott, M. D. A Manual of Diseases of the Eye, by Edward Jackson, A. M., M.D.

The International Text-book will present a complete treatise on the theory and practice of surgery in its most advanced aspects. There is a real need among practitioners and advanced students for a work on surgery, encyclopedic in scope, yet so condensed in style and arrangement that the matter usually diffused through four or five volumes shall be given in one-half the space and at a correspondingly moderate cost.

In his Pelvic Inflammations, Dr. Pryor directs the attention of the general practitioner and specialist to a surgical treatment of the infectious pelvic diseases of women. The subject is a most important one, inasmuch as inflammatory lesions constitute the majority of all pelvic diseases.

Kyle on the Nose and Throat, Heisler's Embryology, and Jackson's Diseases of the Eye are practical text-books for students, written by men of long and successful experience as teachers of these branches.

Special features of Dr. Kyle's book are the logical classification of the diseases, the modern pathology illustrated with new and original cuts, and the extended consideration given to details of treatment.

Abbott on Transmissible Diseases, is an important and timely contribution to the literature of preventive medicine.

---

THE Arlington Chemical Company, Yonkers, N. Y., has sent out to the medical profession a beautiful engraving in antique design of the Hippocratic oath. It is needful that every physician should become familiar with this medical classic, even though the necessity for the administration of such an oath is now obsolete. The Arlington Company will gladly supply any physician who has been overlooked, or whose engraving has been lost in transmission through the mail, upon the receipt of a postal card request addressed to the company at Yonkers, N. Y.

---

MESSRS. W. R. WARNER & CO., of Philadelphia, New York and Chicago, are distributing free to doctors and druggists, a very complete list of drugs, giving apothecary and metric doses. They are arranged in convenient columns and printed on coated linen cloth, size 22x14, for hanging at the prescription counter or in the doctor's office for ready reference. It will be sent to any doctor or druggist upon request by postal card.

---

THE Medical Libraries Fund is raising to enable the editor of *Medical Libraries*, Dr. C. D. Spivak, of Denver, to continue his work of collecting statistical data concerning the existing medical libraries the world over, and to lay the foundation of a rational system of exchange of duplicate books and periodicals between libraries. The request is sent out for all friends of medical libraries to become subscribers to the fund and to send their subscriptions to the treasurer, Dr. Edward Jackson, McPhee Building, Denver, Col.

The Mellier Drug Company, of St. Louis, has recently mailed to the entire medical profession of the United States a handsome engraving of "The First Meeting of the Medical Society of London, held in 1773," together with a circular mentioning by name the members whose portraits are presented in the picture and stating in what particular field each was distinguished.

This engraving should prove an interesting and attractive addition to the office walls, and if through an oversight any physician failed to receive a copy or if his copy was damaged in transit, one can be obtained gratis by applying to the Mellier Drug Company, 2112 Locust street, St. Louis, Mo.

